# The comparison of totally ultrasound-guided versus fluoroscopy-guided shockwave lithotripsy in renal stone treatment: a systematic review and meta-analysis

**DOI:** 10.12688/f1000research.157981.1

**Published:** 2025-02-10

**Authors:** Retta Catherina Sihotang, Nur Rasyid, Ponco Birowo, Gerhard Reinaldi Situmorang, Widi Atmoko

**Affiliations:** 1 Department of Urology, Faculty of Medicine , Cipto Mangunkusumo Hospital, Universitas Indonesia, Jakarta Pusat, DKI Jakarta, 10430, Indonesia

**Keywords:** Shockwave lithotripsy, ultrasound-guided, fluoroscopy-guided, stone-free rate, complication

## Abstract

Extracorporeal shock wave lithotripsy (SWL) has been a well-known therapy since years ago, especially for renal stones less than 20 mm. This study compared the effectiveness of totally ultrasound-guided (US-guided) and fluoroscopy-guided (FS-guided) SWL in treating renal and ureteral stones. A protocol has been registered in PROSPERO databases for systematic reviews. A systematic literature search was conducted in five online databases (PubMed, ScienceDirect, EMBASE, ProQuest, and Scopus). We included all available articles that compared the effectiveness and safety of US-SWL to FS-SWL. A risk of bias assessment was done using Risk of Bias (Rob) Tools for randomized interventional studies and Risk of bias in non-randomized studies interventions (ROBINS-I) Tools for Non-randomized studies. The primary outcome was the stone-free rate, and the secondary outcome was the complication rate. Subgroup analyses were performed for adult and pediatric groups. A comprehensive literature search identified seven comparative articles that matched the criteria: two randomized trials and six retrospective cohort studies comprising 1,255 patients (609 using US-SWL). The results revealed a significant difference in overall stone-free rates between US-guided and FS-guided SWL RR 0.76(95% CI; 0.61-0.95, p=0.02) and in adults RR 0.76(95% CI; 0.60-0.96), but not children groups RR 0.68(95% CI; 0.24-1.88). US-SWL might be favourable due to the radiation-free procedure and real-time presentation. Complication rates were low, and no life-threatening complications were reported. In conclusion, US-guided SWL is more effective than FS-guided SWL for treating renal stones, with a low incidence of complications. Further randomized controlled trials with larger populations are needed to explore the comparison more accurately.

## Introduction

Extracorporeal shock wave lithotripsy (SWL) has been a well-known therapy for treating urinary stones since the early 1980s. ^1, 2^ For the treatment of renal or pyelonephritis stones, there is a current trend toward using minimally invasive endoscopic methods, such as ureteroscopy percutaneous nephrolithotomy. SWL remains one of the leading treatment choices for renal stones less than 20 mm despite this progression. SWL has a low incidence of complications and does not necessitate general anesthesia. ^3– 5^

Ultrasonography (US) (B-scan ultrasound) or fluoroscopy (FS) must be used to appropriately visualize the stone to focus the shock waves as precisely as possible for SWL to be successful (X-rays). Radiopaque stones in the kidney calyces, renal pelvis, or ureteropelvic junction (UPJ) are frequently visible on US and FS. Although the energy source and coupling devices have changed little in recent years, advances in SWL technology have led to the ultrasonic (US) localization of stones. The combination of US and FS has improved the success rate of SWL in a few studies. By alternating ultrasound and fluoroscopy, the lithotripter’s energy can be more concentrated on the target stone throughout the entire session, enhancing SWL efficiency. Unfortunately, few studies have examined the effectiveness of totally ultrasound-based and fluoroscopy-based lithotripters. It is difficult to compare the efficacy between different institutions due to variabilities in treatment procedures and operators. ^6^ This study aims to compare the effectiveness of totally ultrasound-guided (US-SWL) to fluoroscopy-guided shockwave lithotripsy (FS-SWL) in renal stones.

## Methods

This study was conducted on the guideline of Preferred Reporting Items for Systematic Review and Meta-analyses (PRISMA) 2020. The study protocol was registered in the PROSPERO database ( www.crd.york.ac.uk/prospero) under registration number CRD42023403319.

### Search strategy

A comprehensive literature search was conducted in five international online databases: PubMed ( https://pubmed.ncbi.nlm.nih.gov), ScienceDirect ( https://www.sciencedirect.com), EMBASE ( https://www.embase.com), Proquest ( https://www.proquest.com), and Scopus ( https://www.scopus.com) on June 16th, 2024. The keywords used in the search strategy were “ultrasound”, “fluoroscopy”, and “shockwave lithotripsy”. We included all comparative studies, including randomized controlled trials (RCT), prospective non-randomized trials, cohort, and case-control studies comparing the stone-free rate of US-SWL versus the conventional FS-SWL We included all articles that were available in English.

### Eligibility criteria

The included studies’ inclusion criteria were as follows: 1) patients with renal stones; 2) studies comparing totally US-SWL to FS-SWL; 3) reporting stone-free rate and complication rate, if available; 4) Articles available in English. The exclusion criteria were 1) non-comparative studies, 2) meta-analysis studies, 3) review studies; and 4) studies using the combination of US-SWL and FS-SWL.

### Quality assessment

Two reviewers (RCS and NR) screened the available studies independently based on the title and abstracts. The retrieved full texts were reviewed independently to confirm the eligibility criteria and continue to extract data. Each study will be extracted into a table consisting of authors, study designs, subjects, group comparison, location and size of renal stones, stone-free rate, outcome definition, and complication rate. Included articles will be assessed using Risk of Bias (Rob) Tools in Review Manager (RevMan) 5.4 software ( https://revman.cochrane.org) for randomized interventional studies and Risk of bias in non-randomized studies interventions (ROBINS-I) Tools for Non-randomized studies. The quality assessment was performed independently by four contributors (GR, NR, PB, WD).

### Outcomes

The study’s primary outcome was stone-free rate, while the secondary outcome was a complication rate. We also performed subgroup analysis dividing the outcomes in two subgroups, which are adults and children subgroups.

### Statistical analysis

The pooled effect size of dichotomous outcomes was summarized using the risk ratio (RR). The Chi-square test and I2 statistic were used to gauge the degree of study heterogeneity; I2 values below 50% are considered homogenous. In the absence of low heterogeneity, a random effect model was applied. The statistical analyses were performed using Review Manager 5.4. Metanalysis was performed using a forest plot for stone-free and complication-rate outcomes.

## Results

**Figure 1.**
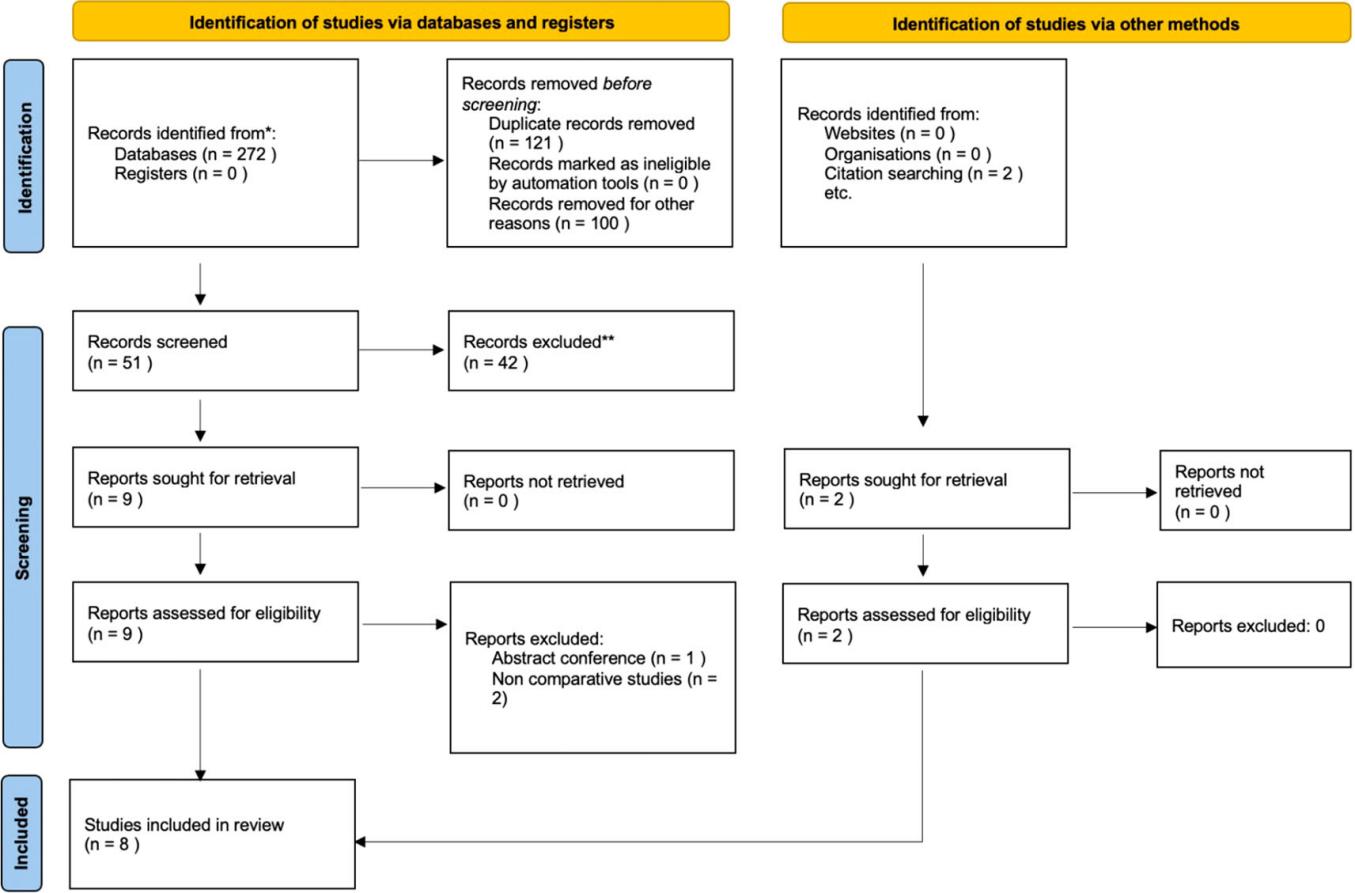
PRISMA flow diagram of study selection.

**Figure 2.**
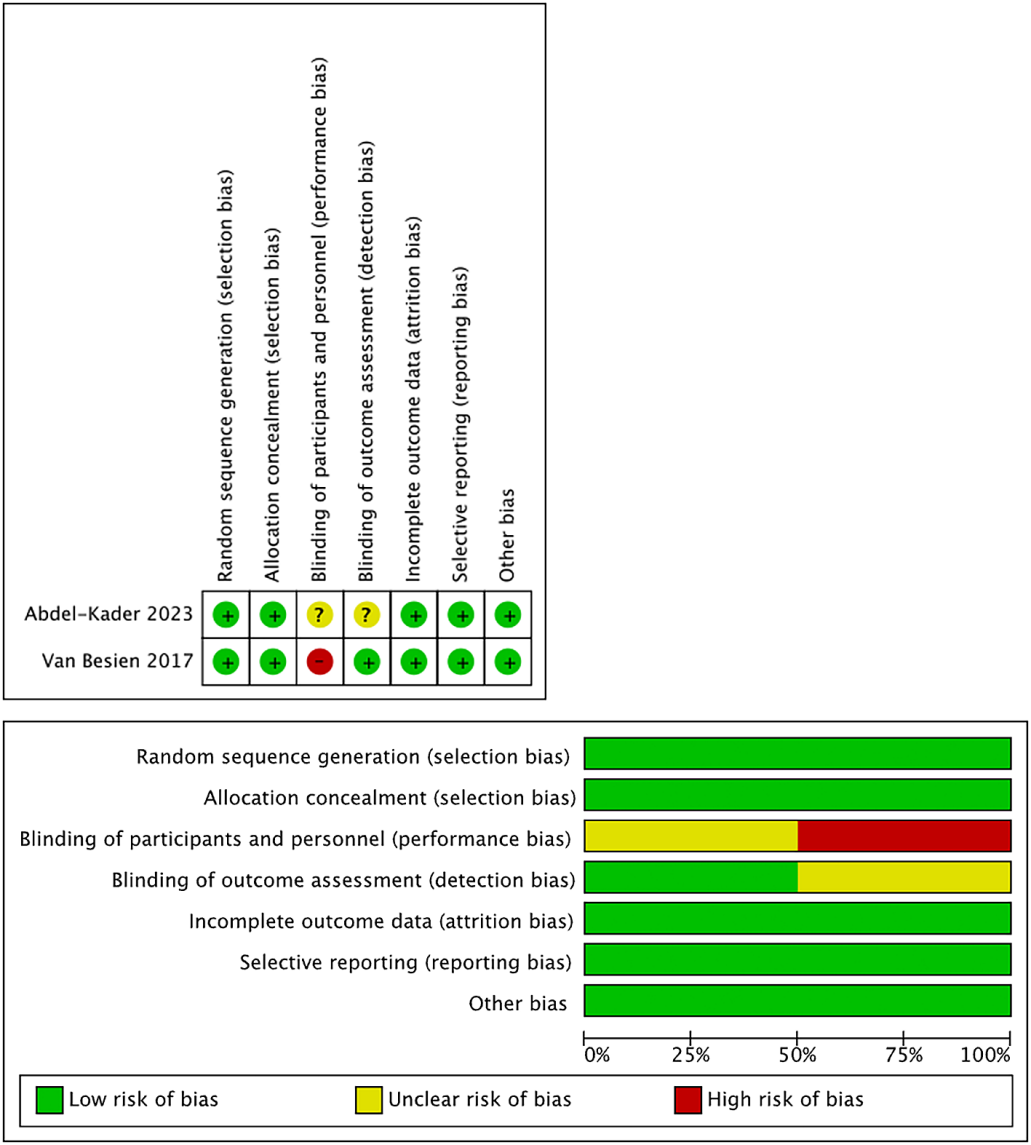
Risk of Bias (Rob) Tool for randomized interventional studies.

We retrieved 272 hits on the five online databases ( Table 1). A total of 121 duplicate articles and 100 irrelevant studies were excluded from the analysis. After screening and selecting the articles, we included eight articles in this systematic review and quantitative analysis ( Figure 1). The articles included two randomized trials and six retrospective cohort studies. ^7– 14^ The characteristics of the included studies are presented in Table 2. We extracted any available data from the studies, including subjects’ characteristics, stone size, stone density (in Hounsfield Unite - HU), stone location, SFR definition, SWL technique, and the outcomes. The quality of the study showed a low to moderate risk of bias based on the Rob Tools and ROBINS-I Tools ( Table 3, Figure 2). The oldest study was conducted in 2010, and the most recent study was conducted in 2023. The sample size varied from 40 to 495 patients and was divided into US-SWL and FS-SWL groups. Most patients presented renal stones from the imaging examination, however, a study by Motolova et al. ^10^ included patients with proximal and distal ureters. We performed a quantitative analysis of seven studies, consisting of three of the children population and four of the adult population. Funnel plot analysis could not be performed because the included studies were less than 10.

**Table 1.** Literature Search Strategies and Results.

Database	Search Query	Result	Access Date
PubMed	(Ultrasound shockwave lithotripsy [Title/Abstract]) AND (fluoroscopy shockwave lithotripsy [Title/Abstract])	38	16 June 2024
ScienceDirect	Ultrasound Shockwave Lithotripsy [Title, abstract, keyword]	35	16 June 2024
EMBASE	(‘ultrasound shockwave lithotripsy’ OR ((‘ultrasound’/exp OR ultrasound) AND (‘shockwave’/exp OR shockwave) AND (‘lithotripsy’/exp OR lithotripsy))) AND (‘fluoroscopy guided’ OR ((‘fluoroscopy’/exp OR fluoroscopy) AND guided))	13	16 June 2024
ProQuest	ultrasound AND fluoroscopy shockwave lithotripsy AND renal stone [Title, abstract, keyword]	167	16 June 2024
Scopus	ultrasound AND fluoroscopy shockwave lithotripsy AND renal stone	19	16 June 2024

**Table 2.** Table of summary for study findings.

Authors	Study design	Subjects	Comparison	Stone free rate
Abdel Kader, 2023 ^7^	Prospective randomized study	**Children** aged 2–16 years who presented with radiopaque renal pelvic stones < 20 mm	**Group US**: 50 patients **Group FS**: 50 patients	After 1 month follow up ( *p* 0.749): **Group US**: 42/50 (84%) **Group FS**: 35/50 (90%)
Arunagiri, 2010 ^8^	Prospective non randomized study (Dissertation)	**Adults** patients with Renal stones 5mm – 2 cm in diameter in the upper, middle calyx or Renal Pelvis and ≤ 1cm in the lower calyx. Mean age 34.74(9.8) years old (Group US) and 31.32(6.8) years old (Group FS)	**Group US**: 50 patients **Group FS**: 50 patients	After 2 weeks: **Group US**: overall 35/50 (70%) <5 mm 4/4 (100%) 6-10 mm 12/17 (70.5%) 11-20 mm 19/29 (65.5%) **Group FS**: overall 32/50 (64%) 6-10 mm 6/10 (60%) 11-20 mm 26/40 (65%)
Goren, 2017 ^9^	Retrospective study	**Children** with renal stones treated between January 2009 and August 2015 were retrospectively reviewed.	**Group US**: 31 patients **Group FS**: 20 patients	Initial SFR: **Group US**: 25/31 (80,6%) **Group FS**: 5/20 (25%) 3 months follow up SFR (p = 0.008): **Group US**: 29/31 (93,5%) **Group FS**: 12/20 (60%)
Motolova, 2021 ^10^	Retrospective study	**Adult** population as the first intervention to solve X-ray-contrast nephrolithiasis, proximal and distal ureterolithiasis of size 6–13 mm	**Group US**: 120 patients **Group FS**: 140 patients	**Group US**: 108/120 (90%) **Group FS:** 126/140 (90%)
Ozkaya, 2019 ^11^	Retrospective study	**Children** under 16 years of age who were treated with SWL using ultrasonic and fluoroscopic focusing were included in the study.	**Group US**: 233 patients **Group FS**: 262 patients	**Group US**: 215/233 (92.3%) **Group FS:** 237/262 (90.5%)
Periasamy, 2024 ^12^	Retrospective Study	**Adults** age 20-60 years old were retrospectively reviwed	**Group US:** 20 patients **Group FS:** 20 patients	**Group US:** 17/20 (85%) **Group FS:** 16/20 (80%)
Smith, 2015 ^13^	Retrospective study	**Adults** patients receiving initial treatment for renal calculi in our unit on the same lithotripsy machine from 2012 to 2013	**Group US:** 48 patients **Group FS**: 47 patients	**Group US:** 29/48 (60%) < 7mm 18/21(85.7%) >7mm 11/27 (40.7%) **Group FS**: 21/47 (45%) <7mm 10/17(58.5%) >7mm 11/30(36.6%)
Van Besien, 2017 ^14^	Randomized Prospective	Patients with radiopaque UUTS were eligible to be enrolled in this prospective single-center study.	**Group US**: 57 patients **Group FS**: 57 patients	**Group US**: 34/57 (52%) **Group FS**: 24/57 (42%)

FS=Fluoroscopy; LUTS=Lower Urinary Tract Symptoms; US=ultrasound.

**Table 3.** Risk of bias in non-randomized studies interventions (ROBINS-I) Tools for Non randomized studies.

Study	Bias due to confounding	Bias in selection of participants into the study	Bias in classification of interventions	Bias due to deviations from intended interventions	Bias due to missing data	Bias in measurement of outcomes	Bias in selection of the reported result	Overall bias
**Arunagiri 2011**	Moderate	Moderate	Low	Low	Low	Moderate	Moderate	Moderate
**Goren 2017**	Low	Moderate	Low	Low	Low	Low	Low	Moderate
**Motolova 2021**	Low	Moderate	Low	Low	Low	Moderate	Low	Moderate
**Ozkaya 2019**	Low	Low	Low	Low	Low	Low	Low	Low
**Periasamy 2024**	Moderate	Low	Low	Low	Low	Low	Low	Low
**Smith 2016**	Moderate	Moderate	Low	Low	Low	Moderate	Low	Moderate

### Stone free rate

**Figure 3.**
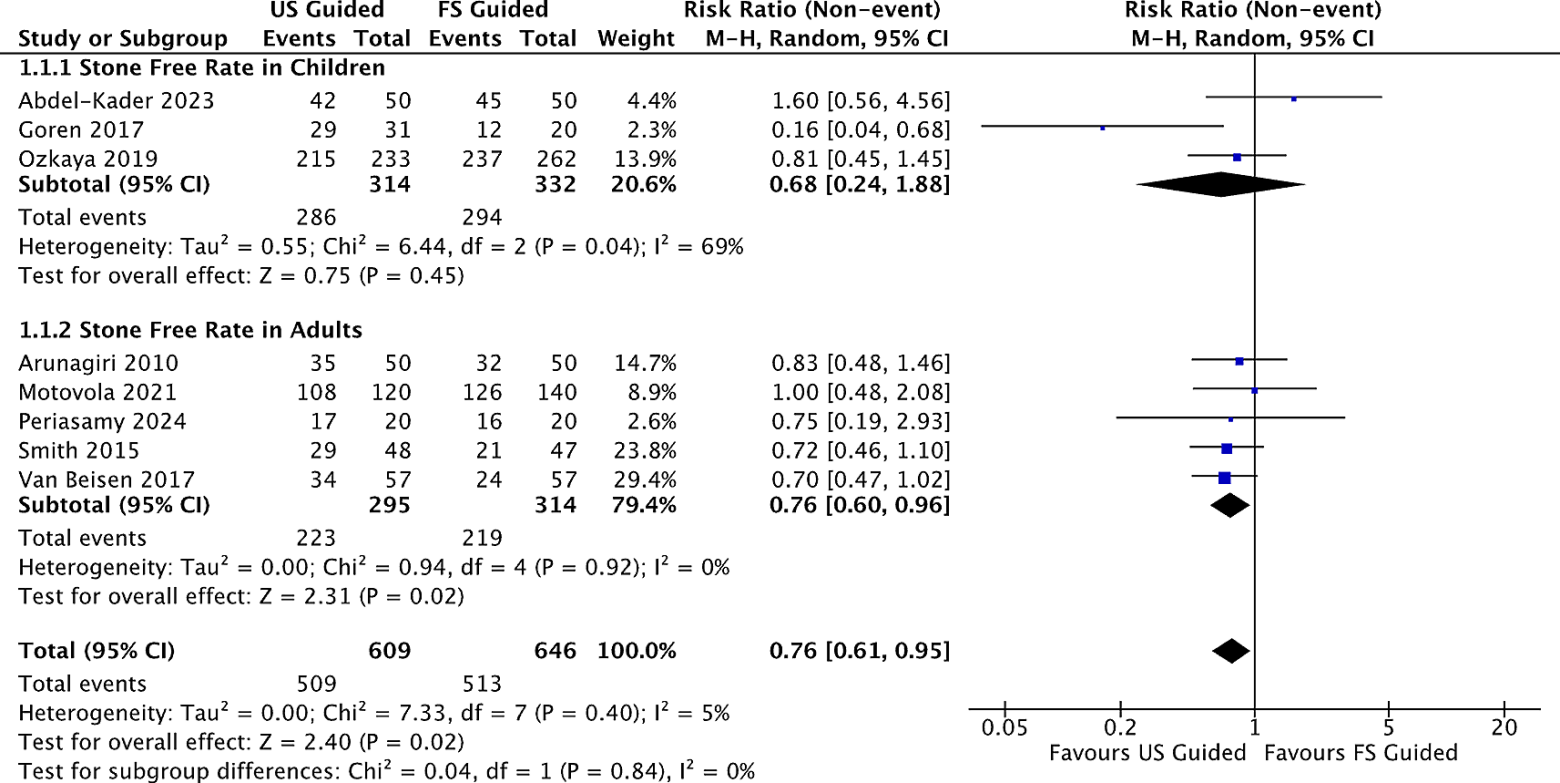
Forrest plot of stone free rate.

The stone-free rate is an absence of residual stones in a follow-up period after the shockwave lithotripsy procedure. The stone-free rate definition varied across studies, which might be a confounding factor in the result. Eight studies comprised 1,255 patients; 609 (48,5%) underwent ultrasound shockwave lithotripsy. The stone-free rate in US-SWL varied from 52-93%, while in FL-SWL varied from 40-90.5%. In our metaregression analysis, there were significant differences observed in the case SFR between ultrasound-guided and fluoroscopy-guided RR 0.76(95% CI; 0.61-0.95, p=0.02) ( Figure 3). We also divided the patients into adult RR and children groups. There were significant differences within the adult RR 0.76(95% CI; 0.60-0.96) but not significant in children groups children groups RR 0.68(95% CI; 0.24-1.88), respectively.

### Complication rate

**Figure 4.**
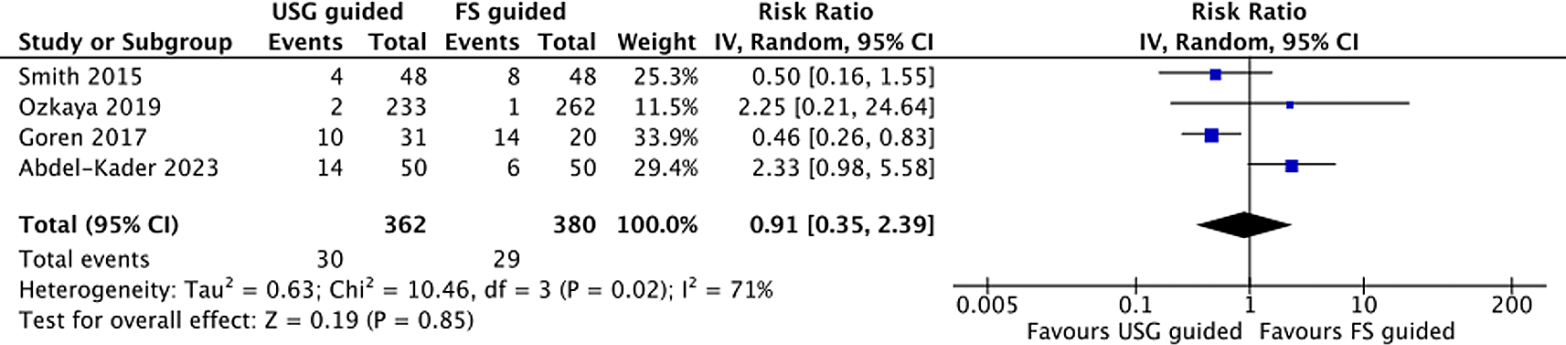
Forrest plot of complication rate.

We included all studies that prove complication rate data. Five studies reported complication rates between the two groups. The reported complications were pain, transient hematuria, fever, urinary tract infection, steinstrasse, and further interventions. However, we exclude one article ^12^ due to outlier values. Of the four studies, three studies reported the incidence in the Clavien-Dindo classification system (grade I - V). One study only reported the complication by the need for further interventions. None of the studies reported complications of Grade IV or V (threatening complications). In our analysis, the complication rate between the two groups did not differ by RR 0.91(95% CI; 0.35-2.39) ( Figure 4). The management of complications is mainly pharmacological, using anti-inflammatory drugs and antibiotics, and some need further interventions such as additional SWL sessions, secondary ureteroscopy, or percutaneous nephrolithotomy. A visual presentation of the funnel plot showed no potential sources of small study effects for complication rate ( Figure 4).

## Discussion

US-SWL and FS-SWL employ focused shockwaves to break kidney stones. However, the two techniques differ in their imaging modalities for localizing the stone during treatment. ^15– 17^ Ultrasound is radiation-free, reducing the risk of radiation-induced complications for patients and healthcare professionals. ^18^ Additionally, ultrasound is a real-time imaging modality, allowing for continuous monitoring and adjustment during the procedure and providing an accurate assessment of stone fragmentation and size. Focusing the shockwaves on the stone can be accomplished to achieve optimal fragmentation. ^9, 19^ On the other hand, FS-SWL relies on fluoroscopy for stone localization. ^20^ Despite the exposure to ionizing radiation, fluoroscopy can better visualize certain stone types, particularly those with high radiopacity. This can lead to improved treatment outcomes in specific cases. ^21^

This is the first meta-analysis comparing the effectiveness (SFR) and safety (complication rates) of US-SWL and FS-SWL. For the stone-free rate outcome, we divided the analysis based on the population, adults and children. In our study, we found that US-SWL is more effective than FS-SWL in terms of stone-free rate after the procedure. The stone-free rate ranged from 52 to 93% for the US-SWL group and 40 to 90.5% for the FS-SWL groups. Studies from Goren et al., ^10^ Arunagiri et al., ^8^ Smith et al., ^13^ and Van Beisen et al. ^14^ showed a higher stone-free rate in the US-SWL group than the FS-SWL group. However, in the children population analysis, the stone-free rate does not differ. This might be due to the cooperativeness during the procedure.

Similar results were also demonstrated in the complication rate. Of the seven studies, only four studies provide the measurement of complication rates. The complication was relatively low, ranging from 0,1-32%. A study by Goren et al. ^10^ reported significantly lower complications in the US-SWL group, while other studies reported no significant difference. None of the complications was life-threatening. There were no significant differences between both groups.

The reported complications were pain, lower urinary tract symptoms, transient hematuria, fever, urinary tract infection, steinstrasse, and further interventions. The management of the complications was mainly conservative with supported medication (anti-inflammatory, analgesic, antibiotics). Subjects with Clavien Dindo Grade 3 required further intervention for ureteric stenting or endourology procedures.

Generally, numerous factors can impact the ultimate stone-free rate (SFR) outcome of extracorporeal shock wave lithotripsy (SWL), regardless of the guidance method used. The factors were divided into stone characteristics (size, composition, and location), patient characteristics (age, BMI, anatomical factors), and technique-related factors (shockwave energy and frequency, number of treatment sessions, and operator experience). Stone size, density, and locations affect the outcome of SWL. The results of SWL for renal stones up to 10 mm in diameter are satisfactory regardless of their location in the kidney. ^22– 24^ Stone density obtained from CT KUB was demonstrated as a predictor for the success rate of SWL. ^25, 26^ Some stones (e.g., calcium oxalate monohydrate and cystine) are harder and more resistant to fragmentation, leading to a lower SFR. Gupta et al. ^27^ found that SWL outcomes were best when the mean stone density was 750 HU. In a separate prospective study involving 50 patients with urinary stones, the author determined that a stone density threshold of 970 HU is a precise and sensitive predictor of SWL outcome. ^28^ A study by El-Nahas et al. ^29^ discovered that stone density greater than 1000 HU significantly predicts SWL failure. El-Assmy et al. ^30^ concluded that an HU value of 600 HU and a stone length of 1.2 cm were significant independent predictors of SWL efficacy when treating urinary stones in children. Additionally, Stones located in the lower pole of the kidney or lower ureter tend to have a lower SFR due to challenges in clearing the fragments. ^16^

The patient’s age, BMI, and anatomical abnormalities may also alter the SFR. Older patients may have a lower SR due to age-related factors, such as reduced renal function or altered anatomy. ^19^ A higher BMI can decrease the effectiveness of SWL by increasing the distance between the shockwave source and the stone. Waqas et al. found that patients with BMI <30 kg/m2 have a higher SWL success rate than patients with BMI >30 kg/m2. ^31^ Anatomy abnormalities, such as patients with skeletal anomalies, renal malformations, or strictures in the urinary tract, may have a lower SFR due to difficulty reaching the stones and unfavorable fragments passage. ^21^

The SF can be affected by the frequency and energy of the shockwaves, with higher energy and lower frequency generally yield better results. A systematic review and meta-analysis by Kang et al. ^32^ showed that low-frequency success rates (OR 2.2; 95% CI 1.5-2.6) and intermediate-frequency SWL (OR 2.5; 95% CI 1.3-4.6) were higher than high-frequency SWL.

Multiple sessions may be required to achieve a higher SFR. According to Goren et al., ^10^ the median number of SWL sessions in the US-guided group was considerably smaller than in the FL-guided group. In a cohort study by Grabsky et al. ^33^ in the pediatric population, the SFR after only 1- session of SWL was 88.0% and increased to 91.7% after several sessions. Several other studies also reported the high SF in SWL achieved after several treatment sessions. ^34, 35^

SWL treatment’s effectiveness also relies upon the operator’s level of expertise. The requirement of pinpoint imaging localization of the stone and proper acoustic coupling to the flank region of the patient is essential because these factors directly influence the quality of the outcome. ^14^ In children, the US-SWL faces technical challenges. The probe used on the shockwave lithotripter was commonly convex and adult-sized. For focusing stones on infants and children, probes sized for adults can be challenging to use. In patients with a small abdominal volume, pressing the abdomen with an adult-sized probe may shift the kidney toward adjacent organs, resulting in the coaction of surrounding tissues by the shockwaves. ^10^

### Limitation

The current study only assessed stone-free and complication rates as outcomes due to limited and dissimilar data of other secondary outcomes in the studies. Thus, we could not compare the factors that might confound the outcome, such as stone size, stone location, stone density, patient characteristics, and technique-related factors. There was also a varied definition of SFR among studies, thus might be a potential bias in the outcome. Moreover, the studies included in the analysis also consisted of various study designs, mostly retrospective with a relatively small sample size, which might be a potential bias itself. Consequently, the results could be overestimated because of selection bias.

## Conclusions

The current study found significant differences in stone-free rates of renal stone between the US-SWL and FS-SWL in the adult group but not in the pediatric groups. There is no difference in terms of complication rates between the two imaging modalities. None of the studies reported any life-threatening complications. The US-SWL is more effective than FS-SWL in treating renal and ureteral stones, with a low incidence of complications, especially in the adult population. Further randomized controlled trials with larger populations are needed to explore the comparison more accurately. We also recommend evaluating the effectiveness of both modalities in ureteral stones in future studies.

## Ethics and consent

No ethics and consent were required.

## Data Availability

No data are associated with this article. Figshare: “Study Characteristics from Included Studies”, https://doi.org/10.6084/m9.figshare.28263743.v1. ^36^ This project contains following extended data:
• Supplementary Data_Study Characteristics.docx Data are available under the terms of the Creative Commons Attribution 4.0 International license (CC-BY 4.0). Figshare: PRISMA checklist for “The comparison of totally ultrasound-guided versus fluoroscopy-guided shockwave lithotripsy in renal stone treatment: a systematic review and meta-analysis”, https://doi.org/10.6084/m9.figshare.27231654.v1. ^37^ Data are available under the terms of the Creative Commons Attribution 4.0 International license (CC-BY 4.0).

## References

[1] Manzoor H Saikali SW : Renal extracorporeal lithrotripsy. *Statpearls.* 2022. Reference Source 32809722

[2] Chaussy C Brendel W Schmiedt E : Extracorporeally induced destruction of kidney stones by shock waves. *Lancet (London, England).* 1980;2(8207):1265–1268. 10.1016/S0140-6736(80)92335-1 6108446

[3] Shafi H Moazzami B Pourghasem M : An overview of treatment options for urinary stones. *Caspian J. Intern. Med.* 2016;7(1):1–6. 26958325 PMC4761115

[4] Ruggera L Beltrami P Ballario R : Impact of anatomical pielocaliceal topography in the treatment of renal lower calyces stones with extracorporeal shock wave lithotripsy. *Int. J. Urol.* 2005;12:525–532. 10.1111/j.1442-2042.2005.01101.x 15985072

[5] Ghoneim IA Ziada AM Elkatib SE : Predictive factors of lower calyceal stone clearance after extracorporeal shockwave lithotripsy (ESWL): A focus on the infundibulopelvic anatomy. *Eur. Urol.* 2005;48:296–302. 10.1016/j.eururo.2005.02.017 16005376

[6] Chang T-H Lin W-R Tsai W-K : Comparison of ultrasound-assisted and pure fluoroscopy-guided extracorporeal shockwave lithotripsy for renal stones. *BMC Urol.* 2020;20(1):183. 10.1186/s12894-020-00756-6 33172476 PMC7653739

[7] Abdel-Kader MS Fathy A Moubarek M : Which is better, fluoroscopic-guided or ultrasonic-guided shock wave lithotripsy for pediatric renal stones? prospective randomized comparative study. *World. J. Urol.* 2023;1(1):1–6. 10.1007/s00345-023-04313-2 36746808

[8] Arunagiri A : *Comparative study of efficacy of localization and fragmentation of renal stone by USG and fluoroscopy guided ESWL.* Chennai: Kilpauk Medical College;2010. [master’s thesis].

[9] Goren MR Goren V Ozer C : Ultrasound-guided shockwave lithotripsy reduces radiation exposure and has better outcomes for pediatric cystine stones. *Urol. Int.* 2017;98(4):429–435. 10.1159/000446220 27160372

[10] Motolová M Král M : Ultrasound versus fluoroscopic localisation during extracorporeal shockwave lithotripsy. *Ces Urol.* 2021;25(2):112–9. Czech.

[11] Ozkaya F : Comparison of the results of shock wave lithotripsy with ultrasonic and fluoroscopic focus in pediatric age group; fluoroscopic focusing how much is needed? *Ann. Med. Res.* 2021;26(11):2502–2506. 10.5455/annalsmedres.2019.10.600

[12] Periasamy P Narashimman J Maruthamuthu R : The comparison of outcomes between USG guided ESWL Vs Fluoroscopy guided ESWL – Our institutional experience. *Asian J. Med. Sci.* 2024;15(2):227–230. 10.3126/ajms.v15i2.59809

[13] Smith HE Bryant DA KooNg J : Extracorporeal shockwave lithotripsy without radiation: ultrasound localization is as effective as fluoroscopy. *Urol. Ann.* 2016;8(4):454–457. 10.4103/0974-7796.192104 28057991 PMC5100152

[14] Van Besien J Uvin P Hermie I : Ultrasonography is not inferior to fluoroscopy to guide extracorporeal shock waves during treatment of renal and upper ureteric calculi: a randomized prospective study. *Biomed. Res. Int.* 2017;2017:7802672. 28589147 10.1155/2017/7802672 PMC5447263

[15] Sheir K Madbouly K Elsobsky E : Prospective randomized comparative study of the effectiveness and safety of electrohydraulic and electromagnetic extracorporeal shockwave lithotriptors. *J. Urol.* 2003;170:389–392. 10.1097/01.ju.0000075080.58359.46 12853782

[16] Türk C Skolarikos A Neisius A : *EAU guidelines on urolithiasis.* European Association of Urology;2023.

[17] Connors BA Evan AP Willis LR : The effect of discharge voltage on renal injury and impairment caused by lithotripsy in the pig. *J. Am. Soc. Nephrol.* 2000;11(2):310–318. 10.1681/ASN.V112310 10665938

[18] Smith-Bindman R Moghadassi M Wilson N : Radiation dose associated with common computed tomography examinations and the associated lifetime attributable risk of cancer. *Arch. Intern. Med.* 2009;169(22):2078–2086. 10.1001/archinternmed.2009.427 20008690 PMC4635397

[19] Chaussy C Schmiedt E Jocham D : First clinical experience with extracorporeally induced destruction of kidney stones by shock waves. *J. Urol.* 1982;127(3):417–420. 10.1016/S0022-5347(17)53841-0 6977650

[20] Lingeman JE McAteer JA Gnessin E : Shock wave lithotripsy: advances in technology and technique. *Nat. Rev. Urol.* 2009;6(12):660–670. 10.1038/nrurol.2009.216 19956196 PMC2923385

[21] Zhu W Liu Y Liu L : Ultrasound versus fluoroscopy-guided percutaneous nephrolithotomy: a systematic review and meta-analysis. *Urolithiasis.* 2018;46(5):431–443.

[22] Lingeman JE Siegel YI Steele B : Management of lower pole nephrolithiasis: a critical analysis. *J. Urol.* 1994;151:663–667. 10.1016/S0022-5347(17)35042-5 8308977

[23] Ilker Y Tarcan T Akdas A : When should one perform shock wave lithotripsy for lower caliceal stones? *J. Endourol.* 1995;9:439–441. 10.1089/end.1995.9.439 8775070

[24] Chen RN Streem SB : Extracorporeal shock wave lithotripsy for lower pole calculi: long-term radiographic and clinical outcome. *J. Urol.* 1996;156:1572–1575. 10.1016/S0022-5347(01)65450-8 8863540

[25] Abdelaziz H Elabiad Y Aderrouj I : The usefulness of stone density and patient stoutness in predicting extracorporeal shock wave efficiency: results in a North African ethnic group. *Can. Urol. Assoc. J.* 2014;8(7–8):E567–E569. 10.5489/cuaj.1849 25210567 PMC4137029

[26] Magnuson WJ Tomera KM Lance RS : Hounsfield unit density accurately predicts ESWL success. *Alaska Med.* 2005;47(2):6–9. 16459477

[27] Gupta NP Ansari MS Kesarvani P : Role of computed tomography with no contrast medium enhancement in predicting the outcome of extracorporeal shock wave lithotripsy for urinary calculi. *BJU Int.* 2005;95(9):1285–1288. 10.1111/j.1464-410X.2005.05520.x 15892818

[28] Murshidi MS : Simple radiological indicators for staghorn calculi response to ESWL. *Int. Urol. Nephrol.* 2006;38(1):69–73. 10.1007/s11255-005-3156-y 16502055

[29] El-Nahas AR El-Assmy AM Mansour O : A prospective multivariate analysis of factors predicting stone disintegration by extracorporeal shock wave lithotripsy: the value of high-resolution noncontrast computed tomography. *Eur. Urol.* 2007;51(6):1688–1693. discussion 93-4. 10.1016/j.eururo.2006.11.048 17161522

[30] El-Assmy A El-Nahas AR Abou-El-Ghar ME : Kidney stone size and Hounsfield units predict successful shockwave lithotripsy in children. *Urology.* 2013;81(4):880–884. 10.1016/j.urology.2012.12.012 23395121

[31] Waqas M Ayaz Khan M Waqas Iqbal M : Non-contrast computed tomography scan based parameters of ureteric stones affecting the outcome of extracorporeal shock wave lithotripsy. *Cureus.* 2017;9(5):e1227. 10.7759/cureus.1227 28589076 PMC5459557

[32] Kang DH Cho KS Ham WS : Comparison of high, intermediate, and low frequency shock wave lithotripsy for urinary tract stone disease: Systematic Review and network meta-analysis. *PLoS One.* 2016;11(7):e0158661. 10.1371/journal.pone.0158661 27387279 PMC4936716

[33] Grabsky A Tsaturyan A Musheghyan L : Effectiveness of ultrasound- guided shockwave lithotripsy and predictors of its success rate in pediatric population: a report from a national reference center. *J. Pediatr. Urol.* 2021;17(1):78.e1–78.e7. 10.1016/j.jpurol.2020.10.014 33153916

[34] D’Addessi A Bongiovanni L Sasso F : Extracorporeal shockwave lithotripsy in pediatrics. *J. Endourol.* 2008;22(1):1–12. 10.1089/end.2007.9864 18177237

[35] He L Sun X Lu J : Comparison of efficacy and safety of shockwave lithotripsy for upper urinary tract stones of different locations in children: a study of 311 cases. *World. J. Urol.* 2011;29(6):713–717. 10.1007/s00345-010-0592-9 21153828

[36] Sihotang RC Rasyid N Birowo P : PRISMA CHECKLIST_The comparison of totally ultrasound-guided versus fluoroscopy-guided shockwave lithotripsy in renal stone treatment: a systematic review and meta-analysis.[Dataset]. *figshare.* 2024. 10.6084/m9.figshare.27231654.v1 PMC12511851 41079303

[37] Sihotang RC Birowo P Rasyid N : Study Characteristics from Included Studies.Dataset. *figshare.* 2025. 10.6084/m9.figshare.28263743.v1

