# Supplementary material for: The comparison of totally ultrasound-guided versus fluoroscopy-guided shockwave lithotripsy in renal stone treatment: a systematic review and meta-analysis
Source: F1000Res. 2025 Jul 7;14:181. Originally published 2025 Feb 10. [Version 2] doi: 10.12688/f1000research.157981.2 (PMC12511851; doi:10.12688/f1000research.157981.2)
Supplement: Peer review history

# Reviewer report: Reviewer response for version 2

**DOI:** 10.5256/f1000research.184222.r416537

**Published:** 2025-10-09

**Authors:** Yu-Ren Huang

**Affiliations:** 1 Case Western Reserve University, Cleveland, Ohio, USA

Overall, I find the manuscript is well written and suitable for indexing.

**Minor Revisions:**

- In some of the studies listed, the location and size of the stones were missing per table 2 shown. The author indicated the limitation of the studies, however, if the subgroups of stones can be analyzed, it would be helpful to address the heterogeneity and bias.
- The baseline characteristics of the included studies should be described in more detail. It would be valuable to include variables such as the hospital setting,  patient ethnicity, geographical background and the stone related history to provide better context of the participants.
- The manuscript currently reports complication rates without clearly differentiating whether these complications are directly attributable to the extracorporeal shock wave lithotripsy procedure or are sequelae related to the underlying stone disease (e.g., symptoms from stone passage or pre-existing infections).
- Consider including the specific timeframe for included studies (e.g., from year X to 2024) for completeness.

Are the rationale for, and objectives of, the Systematic Review clearly stated?

Partly

Is the statistical analysis and its interpretation appropriate?

Yes

If this is a Living Systematic Review, is the ‘living’ method appropriate and is the search schedule clearly defined and justified? (‘Living Systematic Review’ or a variation of this term should be included in the title.)

Not applicable

Are sufficient details of the methods and analysis provided to allow replication by others?

Yes

Are the conclusions drawn adequately supported by the results presented in the review?

Partly

Reviewer Expertise:

Urology, Genetics

I confirm that I have read this submission and believe that I have an appropriate level of expertise to confirm that it is of an acceptable scientific standard.

# Reviewer report: Reviewer response for version 2

**DOI:** 10.5256/f1000research.184222.r413988

**Published:** 2025-10-08

**Authors:** Phyllis Yan

**Affiliations:** 1 University of Michigan, Ann Arbor, Michigan, USA

This is an interesting review that highlights the need for additional research to compare the use of ultrasound-guided and fluoroscopy-guided shock wave lithotripsy in treating renal and ureteral stones. I have reviewed this from a statistical perspective and identified some areas for clarity and improvement.  Many of my comments are based on the latest guidelines for meta-analyses and systematic reviews in urology [1].  The comments are as follows:

**Major comments:**

1. Random effects analysis requires an estimate of the between study variance, and this estimate is unreliable when the number of studies is small [1]. Since the number of included studies was less than ten, the authors should use a more conservative approach such as the Hartung-Knapp-Sidik-Jonkman method to calculate the confidence intervals.
2. As other reviewers have noted - for the analyses related to stone free rates in children and complication rates, there seems to be a large amount of heterogeneity and indeed different direction of effects between the studies, although most studies do not have significant results.  Because of this, I think it makes more sense to just describe the potential sources of heterogeneity between the studies rather than to pool the results of the studies, see guidelines 4.1 and 4.2 [1].
3. The risk of bias for each study was assessed, but not incorporated into the findings or conclusions, see guideline 3.3 [1].  Perhaps the authors can assess the quality of evidence of the pooled results using the GRADE approach [2]. 
4. For the stone free rate analyses, I am not sure why the authors chose to present the risk ratio of a non-event, rather than the risk ratio of a stone free event.  I think it would be more intuitive for the authors to present the stone-free risk ratios rather than the non-event risk ratios, and state that the overall stone free rate for US-guided SWL was higher than the stone free rate for FS-guided SWL.  Either way, the authors need to clearly interpret the risk ratios throughout the text, instead of only stating that they reveal a significant or non-significant difference in overall stone-free rates between US-guided and FS-guided SWL.  The authors should describe what event is being modeled, and what is the direction of the effect of US-guided SWL compared to FS-guided SWL.        

**Minor comments:**

1. In the abstract the authors state that “A comprehensive literature search identified seven comparative articles that matched the criteria”, should this be eight?  Similarly, in the results section, the authors state “We performed a quantitative analysis of seven studies, consisting of three of the children population and four of the adult population”.  However, in the forest plot there seem to be five studies included for the adult population. 
2. Quality assessment: How were disagreements in data abstraction and risk of bias assessment handled?
3. PRISMA figure: For the statements “Records identified from*”, and “Records excluded**”, what are the asterisks referring to?
4. Statistical analysis: The statistical analysis section needs to be expanded.  All the analyses performed in the study should be described here, including any subgroup analyses, and the meta-regression mentioned in the “Stone free rate” section.  The authors should also state what covariates were included in the meta-regression.
5. Statistical analysis: The authors should state the estimation method(s) used for the random effects models.
6. Statistical analysis: The authors should report the tau ^2^ statistic rather than the I ^2^ statistic to assess heterogeneity, see guideline 4.3 [1].
7. Statistical analysis: The authors state that “Metanalysis was performed using a forest plot”.  Metanalysis should be changed to “Meta-analysis”, and a forest plot is used to display the results from a meta-analysis, not perform the meta-analysis.
8. Results: The abbreviation SFR should be introduced as “stone free rate (SFR)”.
9. Stone free rate: The authors should elaborate on how the stone-free rate definition varied across studies or cite their supplementary data.
10. Complication rate: The authors should elaborate on why they excluded one study due to outlier values.  Which variable had outlier values?
11. Complication rate: I do not see the visual presentation of the funnel plot, and the referenced figure 4 is the forest plot of the complication rate.  Also, why not present a visual funnel plot for the stone free rate outcome, if there is a funnel plot for the complication rate outcome?
12. Figures 3 and 4: The labels should say “Forest” rather than “Forrest”.
13. Figures 3 and 4: It would be helpful to have the number of total events for US and FS shown in the “Subtotal (95% CI)” row, rather than the row below.

Are the rationale for, and objectives of, the Systematic Review clearly stated?

Yes

Is the statistical analysis and its interpretation appropriate?

Partly

If this is a Living Systematic Review, is the ‘living’ method appropriate and is the search schedule clearly defined and justified? (‘Living Systematic Review’ or a variation of this term should be included in the title.)

Not applicable

Are sufficient details of the methods and analysis provided to allow replication by others?

Partly

Are the conclusions drawn adequately supported by the results presented in the review?

Partly

Reviewer Expertise:

Statistics

I confirm that I have read this submission and believe that I have an appropriate level of expertise to confirm that it is of an acceptable scientific standard, however I have significant reservations, as outlined above.

## References

[1] : Guidelines for meta‐analyses and systematic reviews in urology. *BJU International* .2025; 10.1111/bju.16864 10.1111/bju.16864 40920036

[2] : GRADE: an emerging consensus on rating quality of evidence and strength of recommendations. *BMJ* .2008;336(7650) : 10.1136/bmj.39489.470347.AD 924-926 10.1136/bmj.39489.470347.AD PMC233526118436948

# Reviewer report: Reviewer response for version 1

**DOI:** 10.5256/f1000research.173510.r377157

**Published:** 2025-04-14

**Authors:** Niwanda Yogiswara

**Affiliations:** 1 Airlangga University, Surabaya, Indonesia

The manuscript is generally well-written, with an appropriate length and adequate language use. The methodology is acceptable, and it is commendable that the authors have registered their review protocol in PROSPERO.

 Overall, I find the manuscript suitable for acceptance. However, a few minor revisions are necessary to enhance manuscript clarity.

**Minor Revisions:**

- The baseline characteristics of the included studies should be described in more detail. It would be valuable to include variables such as the mean age of participants, gender distribution, hospital setting, and patient ethnicity or geographical background to provide better context of the participants.
- Given the operator-dependent nature of ultrasound, the reviewer suggests the authors specify who performed the ultrasound-guided SWL in the included studies — for instance, whether it was a urologist, a radiologist, or a trained nurse.
- There appears to be notable heterogeneity in the outcomes related to SFR and complication rates. The authors might consider exploring additional sources of this heterogeneity through subgroup analyses beyond age group — for example, by stratifying according to stone location (e.g., upper pole, renal pelvis), the number of sessions, or variations in energy and frequency settings used during shockwave lithotripsy.
- In terms of complications, it is unclear whether the analysis refers to overall complications or specific categories. If feasible, a subgroup analysis for individual complications (e.g., hematuria, steinstrasse) would provide valuable insights. If such analysis cannot be performed, the authors should consider addressing this as a limitation.

Are the rationale for, and objectives of, the Systematic Review clearly stated?

Yes

Is the statistical analysis and its interpretation appropriate?

Yes

If this is a Living Systematic Review, is the ‘living’ method appropriate and is the search schedule clearly defined and justified? (‘Living Systematic Review’ or a variation of this term should be included in the title.)

No

Are sufficient details of the methods and analysis provided to allow replication by others?

Yes

Are the conclusions drawn adequately supported by the results presented in the review?

Yes

Reviewer Expertise:

Urology

I confirm that I have read this submission and believe that I have an appropriate level of expertise to confirm that it is of an acceptable scientific standard.

# Reviewer report: Reviewer response for version 1

**DOI:** 10.5256/f1000research.173510.r369552

**Published:** 2025-04-02

**Authors:** Mohammed Bassil Ismail

**Affiliations:** 1 University of Baghdad, Baghdad, Baghdad Governorate, Iraq

There is substantial heterogeneity in stone-free rates among adults and complication rates, which the authors did not evaluate.

 • Publication bias was not assessed, specifically using a funnel plot and Egger’s test.

 • The authors did not provide key details on stone characteristics, including location and Hounsfield units (HFU), as well as intervention settings of the included studies.

 • I suggest conducting subgroup analyses by stone size and other relevant variables to explore potential sources of heterogeneity.

 • Due to these methodological limitations, rejection is advised.

Are the rationale for, and objectives of, the Systematic Review clearly stated?

No

Is the statistical analysis and its interpretation appropriate?

Yes

If this is a Living Systematic Review, is the ‘living’ method appropriate and is the search schedule clearly defined and justified? (‘Living Systematic Review’ or a variation of this term should be included in the title.)

Not applicable

Are sufficient details of the methods and analysis provided to allow replication by others?

No

Are the conclusions drawn adequately supported by the results presented in the review?

Partly

Reviewer Expertise:

urology

I confirm that I have read this submission and believe that I have an appropriate level of expertise to state that I do not consider it to be of an acceptable scientific standard, for reasons outlined above.
